# Beyond the Cardiorenal Syndrome: Pathophysiological Approaches and Biomarkers for Renal and Cardiac Crosstalk

**DOI:** 10.3390/diagnostics12040773

**Published:** 2022-03-22

**Authors:** Oana Nicoleta Buliga-Finis, Anca Ouatu, Minerva Codruta Badescu, Nicoleta Dima, Daniela Maria Tanase, Patricia Richter, Ciprian Rezus

**Affiliations:** 1 Department of Internal Medicine, “Grigore T. Popa” University of Medicine and Pharmacy, 700115 Iasi, Romania; oana_finish@yahoo.com (O.N.B.-F.); minerva.badescu@umfiasi.ro (M.C.B.); nicoleta.dima@umfiasi.ro (N.D.); daniela.tanase@umfiasi.ro (D.M.T.); ciprian.rezus@umfiasi.ro (C.R.); 2 Internal Medicine Clinic, “Sf. Spiridon” County Clinical Emergency Hospital, 700111 Iasi, Romania; 3 Department of Rheumatology and Physiotherapy, “Grigore T. Popa” University of Medicine and Pharmacy, 700115 Iasi, Romania; patriciarichter12@yahoo.com; 4 Rheumatology Clinic, Clinical Rehabilitation Hospital, 700661 Iasi, Romania

**Keywords:** cardiorenal syndrome, biomarkers, chronic kidney disease, heart failure, small noncoding RNAs

## Abstract

Cardiorenal syndrome encompasses complex multifactorial facets and carries significant morbidity and mortality worldwide. The bi-directional relationship between the heart and kidneys, where dysfunction in one organ worsens the function of the other, has been the leading motor for research in the last few years. In the pathophysiological process, small noncoding RNAs, epigenetics, vascular growth factors, oxidative stress, hemodynamic factors, and biomarkers play a pivotal role in the development of cardiorenal syndrome. It is therefore important to elucidate all the mechanisms in order to provide diagnostic and treatments tools. This review summarizes the hemodynamic and non-hemodynamic pathways along with biomarkers that could be the next target for diagnosis, treatment, and prognosis in cardiorenal syndrome.

## 1. Introduction

Cardiorenal syndrome represents a group of disorders resulting from the pathological interaction between the kidneys and the heart whereby acute or chronic dysfunction in one organ may induce acute or chronic dysfunction in the other [1].

In 1836 Bright discussed the causal association between cardiovascular risk and chronic kidney disease. The passive renal congestion that results from cardiac dysfunction was named “rein cardiaque” in 1903 by french pathologist F. Justin Collet, while the term “cardiorenal syndrome” (CRS) was used in the 1940s to highlight the bidirectional interactions between the kidneys and heart [2].

Cardiac dysfunction can determine injury on the kidney and reciprocally kidney injury will affect both the circulatory system and the heart. The causal relationship between chronic kidney disease, cardiovascular risk, and heart failure was demonstrated by clinical and epidemiological studies. More than 50% of all patients with chronic kidney disease have a 10- to-20-fold increased risk of cardiovascular disease compared to the same age non-CKD population [3]. The overall prevalence of CKD in a meta-analysis of acute and chronic heart failure populations was 49% (with a prevalence in acute HF—53% vs. chronic HF—42%). AKI was present in 23% to 35% of patients [4].

The cardiorenal syndrome classification has five subtypes that reflect primary and secondary pathology and simultaneous codysfunction secondary to the systemic condition [5]. In 2008, the Consensus Conference of the Acute Dialysis Quality Group proposed a classification of CRS into two main groups: cardio-renal and reno-cardiac [6,7].

In addition Ronco et al. classified cardiorenal syndrome from type 1 to 5:

- Type 1 (acute cardiorenal syndrome): acute decompensated heart failure causing acute kidney failure
- Type 2 (chronic cardiorenal syndrome): chronic heart failure leading to kidney dysfunction
- Type 3 (acute renocardiac syndrome): acute aggravation of kidney function causing heart dysfunction
- Type 4 (chronic renocardiac syndrome): chronic kidney diseasegenerating heart dysfunction/disease
- Type 5 (secondary cardiorenal syndromes): systemic conditions causing a simultaneous dysfunction of the heart and kidneys [1,8].

For CRS to be diagnosed, it is relevant to highlight that both organ crosstalk is involved in acute and chronic pathogenesis and functional or structural abnormalities are present in the two organs (Table 1) [1].

## 2. Classification 

### 2.1. CRS Type 1

Cardiorenal syndrome type 1 includes all conditions in which acute cardiac dysfunction causes acute kidney injury (AKI) [9].

This syndrome represents an intricate condition where inflammatory and neurohormonal aspects create a vicious cycle. Pro-inflammatory cytokines produce apoptosis, oxidative stress, aggravate fluid retention and venous congestion [10]. Conditions such as acute decompensated heart failure, acute coronary syndrome, cardiac surgery, and cardiogenic shock are representative of the etiology of CRS type1 [6,11,12]. 

During CRS, type 1 and especially AKI, toll-like receptors (TLRs) play an important role in influencing inflammatory activation [10]. There are two cell lines that are affected by immune attack in CRS type1: renal tubular epithelium and endothelial cells [5]. Virzì et al. showed that TLR stimulation initiates pathological apoptosis of renal tubular epithelial cells and identified a two-fold increase in caspase-8 levels, a three-fold increase in IL-6, and a ten-fold increase in TNF-alpha levels in incubated monocytes with CRS type 1. Recent studies suggest that TLRs are essential components of immunity and are implicated in various cardiac pathologies such as cardiac failure, ischemic myocardial injury, and acute myocardial infarction. Fluid overload in CRS causes bowel edema which destroys intestinal villi endothelial cells, leading to the release of lipopolysaccharide (LPS) from gut bacteria suggests Colombo et al. and Ronco et al. proposed that low intestinal perfusion in heart failure and cardiorenal syndrome type 1 promotes IL-1, IL-6, and TNF-alpha generation by liberating LPS [10].

NGAL or siderocalin is the first renal marker that could be detected in blood and urine after AKI [13]. Ronco et al. emphasized the importance of NGAL, as a urinary measurement in AKI patients has a sensitivity of 90% and specificity of 99% [14].

### 2.2. CRS Type 2

CRS type 2 occurs when chronic abnormalities in cardiac function lead to kidney injury or dysfunction. Chronic hypoperfusion and renal congestion along with right atrial pressure represent a cornerstone in renal dysfunction of these patients [15].

There are several underlying conditions of CRS-2 such as: heart failure with preserved and reduced ejection fraction, atrial fibrillation, ischemic cardiomyopathy, and congenital heart disease [16]. The importance of CKD in HF patients was examined on 118,465 patients with decompensated heart failure in the ADHERE study. Ninety-one percent had impaired kidney function and 56% had an estimated GFR of 15–69 mL/min/ m^2^ [17].

Diagnosis of CRS 2 is based on Improving Global Outcomes (KDIGO)/Kidney Disease Outcomes Quality Initiative (KDOQI) guidelines: albuminuria and/or GFR < 60 mL/in/1.73 m^2^, or a sustained decrease in GFR > 5 mL/min/1.73 m^2^/year or >10 mL/min/1.73 m^2^/5 years or sustained increase in albuminuria along with suspected congestive heart failure before the onset or progression of CKD [18]. The biomarkers detectable in CRS type 2 are: neutrophil gelatinase-associated lipocalin, cystatin C, kidney injury molecule-1, B-type natriuretic peptide, and miRNA-21 [19,20].

### 2.3. CRS Type 3 

CRS type 3 occurs when AKI contributes to the development of acute HF. There are several common leading causes such as electrolyte disorders, metabolic acidosis, and volume overload. During AKI, the heart and kidneys are closely interconnected by the activation of the sympathetic nervous system, activation of coagulation cascades, and the activation of the RAAS [9].

Inflammation-associated cytokines could be involved in transmitting signals from damaged kidney tissue to the heart resulting in myocardial depression. Because the heart and kidneys have high mitochondrial content, any alteration in mitochondrial morphology plays an important role in CRS 3. Mitochondrial fission is an early process in acute cardiac injury that can lead to cardiomyocyte apoptosis [21]. 

Sumida et al. showed that AKI induced mitochondrial fragmentation in heart tissue by promoting phosphorylation of dynamin-related protein 1 (Drp1) [22]. For the diagnosis of CRS 3, there are many biomarkers that have been proposed: cystatin C, IL-6, IL-18, kidney injury molecule 1, neutrophil gelatinase-associated lipocalin, and Netrin-1 [9].

### 2.4. CRS Type 4

CRS type 4 defines cardiovascular involvement in patients with CKD. Fifty percent of the causes of death in patients with CKD are due to acute cardiac events [9].

GFR is an important independent factor of cardiovascular morbidity and mortality, especially in patients with CKD stages 3b to 4. Recently, kidney disease has been considered an independent risk factor for sudden cardiac death due to cardiac arrhythmias that may be related to electrolyte and volume disturbance seen in patients with CKD. Lipid dysmetabolism, insulin resistance, chronic inflammation, hyperhomocysteinemia, hyperphosphatemia, and secondary hyperparathyroidism contribute to cardiovascular disease in patients with CKD with increasing accumulation of toxins such as: erythropoiesis inhibitors, furans, phenols, beta-2-microglobulin, leptin, polyols, and parathyroid hormone) [15].

Biomarkers such as troponins, homocysteine, natriuretic peptides, C-reactive protein, plasminogen-activator inhibitor type 1, serum amyloid A protein, ischemia-modified albumin increase as GFR declines, but the therapeutic implications are not clearly understood [9].

### 2.5. CRS Type 5 

CRS type 5 is characterized by simultaneous involvement of the heart and kidneys in several clinical conditions such as hepatorenal syndrome, Fabry’s disease, and sepsis. High levels of tumor necrosis factor, IL-1, thromboxane, and prostacyclin are present in patients with septic shock, which may alter coronary autoregulation and endothelial function. AKI occurs in 51% of patients with septic shock and in 20% of critically ill patients. The pathogenesis of septic AKI is explained by inflammatory mediators and hemodynamic factors. There are several characteristic biomarkers: lipopolysaccharide-binding protein, procalcitonin, CRP, IL-6, transforming growth factor beta [15].

## 3. Pathophysiology

Kidney function and heart performance are interconnected and this communication between two different systems involves bidirectional and dynamic pathways [23].

Hypertension, diabetes mellitus, atherosclerosis, and medications are the common risk factors for CRS development [24]. Secondary to the arteriosclerosis process, the aging of the population has been proved to increase the risk of chronic CRS (type 2), CRS type 4, and secondary CRS (type 5) [25].

The incidence of CRS depends on the subtype: acute kidney injury (AKI) occurs in 25% to 33% of acute decompensated heart failure (ADHF). In 60% of ADHF cases, AKI can be diagnosed as an exacerbation of previously diagnosed CKD and in HF, CKD has been reported as a comorbidity in 26% to 63% of patients [26].

Increasing evidence indicates a number of mechanisms and pathophysiological processes that play a significant contribution in the development and evolution of CRS [27,28].

**Figure 1:**
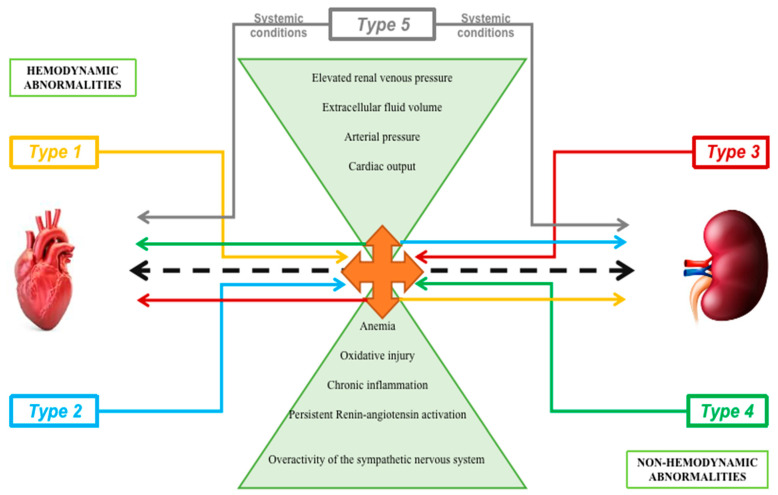
Hemodynamic and non-hemodynamic mechanisms implicated in cardiorenal pathophysiology along with the five types of interaction between the heart and kidney.

In the pathogenesis of cardiorenal syndrome, the multi-factorial mechanisms implicated are not only hemodynamic parameters such as arterial pressure, extracellular fluid volume, and cardiac output but also cell adhesion molecules, endothelial injury, cell death, immunologic imbalance, inflammatory cascades, oxidative stress, neutrophil migration, leukocyte trafficking, cytokine and chemokine overexpression, caspase-mediated induction of apoptotic mechanisms, extracellular vesicles (EVs), small non-coding RNAs, epigenetics, and oxidative stress (Figure 1) [29]. 

Despite the fact that the exact pathophysiology of CRS is unclear, a few mechanisms were proposed: increased central venous and abdominal pressure, reduced cardiac output, oxidative stress, and activation of the renin–angiotensin–aldosterone system (RAAS) [30]. 

### 3.1. Hemodynamic Pathways

Increased intra-abdominal pressure (IAP), intra-abdominal hypertension (IAH), and increased central venous pressure (CVP) are closely correlated to renal function. A retrospective study on patients who underwent right heart catheterization revealed that increased CVP (>6 mmHg) was an independent and strong predictor of all causes of mortality and was also linked to impaired renal function [31]. 

Sixty percent of patients with advanced chronic heart failure have increased IAP (normal values in healthy adults are between 5 and 7 mm Hg). In this group population, an IAP from 8–12 mmHg corresponds to renal dysfunction, leading to cardiorenal syndrome type 2. A decongestive therapy improves serum creatinine levels, reducing abdominal congestion [32].

The two important mechanisms implicated in keeping the renal blood flow constant are: contraction/relaxation of the afferent vessels and the tubule–glomerular feedback. Several animal studies have indicated that a decisive role in renal dysfunction is attributed to an elevation in central venous pressure. Mullens et al. demonstrated that the development of the worsening of renal function in patients with advanced decompensated heart failure was lower for those with a CVP of <8 mmHg. Therefore, venous congestion has an important role in the onset of AKI. An elevated CVP leads to an increase in renal venous pressure (RVP) with consequences in glomerular filtration, worsening the cardiorenal syndrome [33].

Severe depression of cardiac output, in conditions such as cardiogenic shock, leads to acute renal impairment specific for cardiorenal type 1. The activation of the adrenergic cascade in cardiogenic shock may cause ischemic effects (with implications of renal medulla) and also activation of the renin–angiotensin–aldosterone system in order to increase cardiac afterload [34].

Moreover, in patients with heart failure, neurohormonal mechanisms that are activated to restore tissue perfusion such as the overactivity of the sympathetic nervous system result in increased renin levels from juxtamedullary kidney cells [24]. High levels of renin lead to an increased production of angiotensin-2 which has maladaptative effects on the kidneys, heart, and vascularization [35].

The symptoms and signs of hemodynamic abnormalities are probably the most incriminated manifestations of cardiorenal syndrome.

The association between a reduced glomerular filtration rate with heart failure and its worst outcome has been shown in both HF with a reduced ejection fraction and with a preserved ejection fraction. Moreover, tricuspid valve regurgitation was associated with a lower estimated GFR.

Neurohormonal dysregulation and RAAS hyperactivity that are seen in CRS are closely connected to oxidative and inflammatory pathways [36]. Decongestive therapies, neurohormonal modulation, and inhibition of the renin–angiotensin–aldosterone system are major therapeutic strategies for the management of cardiorenal syndromes. Inotropes are beneficial in type 1 CRS, reducing venous congestion. Clinical trials such as CANVAS, DECLARE-TIMI, and EMPA-REG OUTCOME have demonstrated the efficacy of sodium-glucose co-transporter-2 inhibitors (SGLT_2_i): improved glycemic control, reduced intra-glomerular pressure, and reduced intravascular volume. Cardiac resynchronization may improve cardiorenal status in patients with heart failure and CKD [30].

#### 3.1.1. Non-Hemodynamic Pathways

Between the nonhemodynamic pathways that could exacerbate cardiac or kidney injury, the most important include chronic inflammation, activation of the sympathetic nervous system, persistent RAAS activation, and the imbalance in the proportion of reactive oxygen species/nitric oxide production [37]. Particularly in the pathogenesis of cardiorenal syndrome types 1 and 5, an immune-mediated dysregulation could be cited [38].

#### 3.1.2. Endothelial Dysfunction

Endothelial dysregulation has been recognized to contribute to cardiac-renal dysfunction. Therefore, endothelial dysfunction is a critical process in cardiorenal syndrome and has been reported to be associated with type 1 diabetes mellitus, chronic CRS, and type 2 CRS [5].

Endothelial cells are situated at the interface of blood and tissues. These have regulatory activities towards inflammation, vascular homeostasis, and coagulation. They also contribute to immune responses by expressing MHC class I and II antigens [39]. Endothelial dysfunction is described by a systemic modification in vascular wall structure (endothelial cell apoptosis, reduction in endothelial glycocalyx, changes in the basement membrane structure) paralleled with increased vascular permeability, stimulation of a prothrombotic and proinflammatory state, and complement activation.

Risk factors involved in the development of endothelial dysfunction are: smoking, male gender, age, hypertension, dyslipidemia, and lack of physical activity [40]. Endothelial cell activation and dysfunction play an important role in the pathogenesis of cardiorenal syndrome. Regarding the immune responses in CRS, there is evidence that cultured human endothelial cells act as nonprofessional antigen-presenting cells. A smaller number of T cells are activated than those that are activated by professional antigen-presenting cells such as: dendritic cells, macrophages, B cells, and monocytes. There are data that show an important role of professional antigen-presenting cells, nonprofessional antigen-presenting cells, and other immune cells in innate and adaptive immune responses to CRS [5].

An important causal factor for endothelial dysfunction in chronic kidney disease is asymmetric dimethylarginine, an inhibitor of nitric oxide synthase. Ueda et al. proposed a hypothesis to explain the role of asymmetric dimethylarginine in cardiorenal syndrome, explaining the induction of endothelial dysfunction through a pathway of increased endogenous nitric oxide synthase. Primarily endothelial dysfunction induced by asymmetric dimethylarginine contributes to cardiovascular disease. Secondly, endothelial dysfunction could increase asymmetric dimethylarginine via two pathways within the kidney. One pathway is involved in the following sequence: renal ischemia, interstitial fibrosis, glomerular sclerosis, finally leading to chronic kidney disease. Further, the renin–angiotensin system and oxidative stress in chronic kidney disease will determine the dysregulation of dimethylarginine dimethylaminohydrolase (DDAH) and protein arginine methyltransferase (PRMT). The second pathway responsible for increased asymmetric dimethylarginine is proteinuria resulting in the dysregulation of DDAH and PRMT.

Moreover, anti-endothelial cell antibodies, which are known to cause endothelial dysfunction in patients with lupus erythematosus, in type 2 diabetes mellitus, can cause strong endothelial cell contraction and endothelial apoptosis in vitro, contributing to progression to diabetic nephropathy [41].

### 3.2. Epigenetics Implications

In the pathophysiology of heart and kidney disease, exosomes and small non-coding RNAs might participate by transferring genetic material and proteins, mediating intercellular communication, and signaling mechanisms in the target cell. These vesicles may be investigated in acute and chronic kidney damage as molecular indicators of renal structural injury and dysfunction. Moreover, platelets have been shown to release exosomes which may be implicated in the interaction between different cells in atherosclerosis plaques in cardiovascular disease [29].

Extracellular vesicles are cell-derived vesicles with a diameter from 30 to 2000 nm, walled in a lipid bilayer [42,43]. Identification of the content of these EVs in terms of proteome and nucleic acid could offer information on the cell or tissue physiological condition and their origin [44].

It is known that miRNAs are involved in numerous biological processes including cellular differentiation and proliferation cell, hemostasis, apoptosis, and inflammation and are present in the pathophysiology of many other diseases [45]. Small non-coding RNAs regulate the coding expression of proteins genes by biding to the untranslated target region 3 or 5 mRNA, controlling mRNA levels through post-transcriptional mechanisms. miRNAs have been found in biological fluids and in the extracellular space, in a stable condition despite the presence of RNAse [29]. miRNAs in the heart are involved in: apoptosis and myocardial fibrosis, myocyte hypertrophy, cardiac remodeling and regeneration, and ventricular dilatation [46].

MiR-21, one of the most abundant types of miRNAs in human tissues, is associated with different biological functions, such as helping to protect the kidneys in response to inflammation and stress [45]. Moreover, it has been shown that miR-21 plays an important regulatory role in the progression of chronic cardiac and renal disease, and is related to pathological cardiac remodeling and fibrosis of both organs [47].

Recent studies showed that microRNAs have a significant role in atherosclerosis and arterial remodeling mechanisms such as: endothelial dysfunction, monocyte activation, and plaque constitution. Beyond the process of cardiac remodeling and fibrosis in which it is known that miRNAs are involved, Mir-21 can generate cardiac fibrosis via activation of collagen and alpha-smooth muscle actin (a-SMA) protein expression. In the kidneys, high levels of miR-21 lead to increased fibrosis markers through inhibition of Notch2 expression and contributes to the progression of acute and chronic kidney disease. miR-21 can also be transported to other cell types by microvesicles where it induces fibrosis [7].

A new mechanism and therapeutic target in CKD-related CVD could be the miR-21 exported from renal tubular epithelial cells via microvesicles, contributing to the development of cardiomyocyte hypertrophy. Suppression of miR-21 may represent a possible therapeutic option and could improve both cardiac and renal function [47]. Some preclinical data claim that antisense oligonucleotide (ASO)-mediated targeting of miR-21 represents an attractive therapeutic strategy. There are three delivery systems for ASOs currently under investigation packaging into polymer nanoparticles, lipid nanoparticles, and through conjugation with antibodies. Future research and studies are needed to determine dosing, delivery routes, and ASO chemical modifications [48].

#### 3.2.1. Role of Vascular Growth Factors

Vascular growth factors are important both in endothelial function and in the pathophysiology of cardiorenal syndrome in diabetes.

There are five different isoforms of vascular endothelial growth factors: VEGFA, B, C, D, and placental growth factor. Their receptors are predominantly expressed in endothelium, macrophages, and smooth muscle cells. In diabetes, the beneficial effects of VEGFA (synthesis of vasodilatory mediators and promoting endothelial cell survival) are lost and this growth factor became a driver of endothelial dysfunction, inflammation, and vascular disease.

Angpt-1 and Angpt-2, the two representative members of angiopoietins, are a category of vascular growth factors that have an important contribution to vascular expansion. Angpt-1 is a regulator of microvascular tone and vascular stabilization and causes arteriolar vasodilatation by releasing nitric oxide through eNOS stimulation and by regulating vascular endothelial adhesion molecule (VE)-cadherin phosphorylation. Angpt2 is an unfavorable predictor for patients with heart failure and ischemic heart disease and is also associated with the clinical prediction for cardiorenal disease in patients with diabetes. The increased Angpt-2/Angpt-1 ratio in the myocardium favors endothelium apoptosis, inflammation, and is associated with albuminuria as a sign of renal impairment [49].

Anti-VEGFA therapy could be a therapeutic strategy for diabetic kidneys, but studies have shown that an excessive inhibition could promote proteinuria; therefore, inhibitors of VEGF could result in: cardiac ischemia, cardiac dysfunction, and an increase in blood pressure. Modulation rather than inhibition of vascular growth factors and the Angpt-1–2 system could offer some beneficial data for the therapeutic strategy in the diabetic population [49].

#### 3.2.2. Megalin and Cubilin Receptors

Megalin, found at the apical membranes of PTCs, is a glycoprotein and part of the LDL receptor family. Megalin has a role in the reabsorption of glomerular-filtered substances such as low molecular weight proteins and albumin. Dysfunction of megalin is associated with the development of proteinuria/albuminuria in diabetic patients, leading to chronic kidney disease (CKD), end-stage kidney disease, and is highly associated with the development of cardiovascular disease. Recent data demonstrate that there are two forms of megalin in human urine: the full-length form that appears to be less abundant in normal individuals but increased in patients with type 2 diabetes, and the ectodomain form [50].

Cubilin is a 460 kDa peripheral glycoprotein anchored to apical membranes in PTCs. It is involved in the absorption of various protein ligands present in glomerular filtrates (albumin, transferrin, and vitamin D-binding protein). Cubilin was originally identified as the receptor for the intrinsic factor–vitamin B12 complex.

In the early stages of diabetic nephropathy, the altered regulation of megalin expression and its functions, together with the impaired functions of cubilin, must be responsible for the early development of proteinuria/albuminuria [51].

Megalin plays an important role in the internalization and activation of vitamin D and vitamin D-binding protein whose activation occurs predominantly in the kidneys. It also has a key role in cholesterol transport, being the receptor for apoJ/clusterin, which is associated with HLD particles and Lp(a) (an atherogenic particle). As Mii et al. have shown, LDL cholesterol and plasma cholesterol levels are associated with genetic variation in the megalin gene in the Japanese population [52].

#### 3.2.3. Hipoxia Inducible Factors

Oxygen deficiency activates the hypoxia-inducible factor (HIF)-1 gene in healthy subjects to set cells for anaerobic functioning and to stimulate angiogenesis. This regulator of oxygen homeostasis is a heterodimer transcription factor composed of an oxygen-regulated alpha subunit and a beta subunit. There are two HIF-alpha subunits: HIF-1 alpha and HIF-2 alpha [53]. HIF-1a and HIF-2a exert mutually antagonistic effects on inflammation and fibrosis in several organs, including the heart, kidneys, and adipose tissue. Upregulation of HIF-1a in endothelial cells and vascular smooth muscle has been implicated in the pathogenesis of neointimal proliferation, activation of proinflammatory pathways, and thus, the progression of atherosclerosis. HIF-1a and HIF-2a play an important role in the evolution and progression of chronic heart failure, chronic kidney disease, atherosclerotic, and hypertensive vascular disorders. In chronic heart failure, HIF-1 alpha can activate the sympathetic nervous system and can also cause inflammation and hypertrophy in cardiomyocytes.

Resveratrol, a sirtuin-1 activator, inhibits HIF-1 alpha, which reduces fibrosis and inflammation in renal cells and reduces hypoxic injury at the cardiomyocyte level. Sodium-glucose cotransporter 2 (SGLT2) inhibitors also activate sirtuin-1, which has important protective properties on both the heart and kidneys [54].

HIF stabilizer, as a novel anti-anemic therapy, has indications for cardiorenal syndrome, erythropoiesis-stimulating agents-resistant anemia, and malnutrition-inflammation-atherosclerosis syndrome [55].

#### 3.2.4. Oxidative Stress

The role of oxidative injury in CRS can be shown by depressed superoxide dismutase in kidney failure and increased activity of myocardial nicotinamide adenine dinucleotide phosphate oxidase in heart failure [36]. These modifications lead to increased production of reactive oxygen species resulting in oxidative injury to the myocardium and kidneys [56].

The most important free radicals especially in cardiorenal syndrome are the reactive oxygen species (ROS) composed of: superoxide anion radical, hypochlorite, nitric oxide radical, hydroxyl radical, hydrogen peroxide, oxygen singlet, and peroxynitrite radical. They are capable of destabilizing homeostasis by damaging DNA, lipids, proteins, and carbohydrates [57].

Matrix metalloproteinase (MMP) from cardiac fibroblasts is activated by reactive oxygen species [58]. MMP is responsible for the degradation of the extracellular matrix including elastins, matrix glycoproteins, collagens, and tissue remodeling. Due to its central role in tissue remodeling in injury and inflammation, it represents a relevant biomarker, especially in CRS [8].

The heart and kidneys compared to other organs have high mitochondrial content and related oxidative stress can lead to cardiorenal damage [21]. Sumida et al. demonstrated that acute kidney injury induced mitochondrial fragmentation in heart tissue by promoting phosphorylation of dynamin-related protein 1 (Drp1). Oxidative stress and inflammation can induce mitochondrial fission which is considered an early sign that can produce cardiomyocyte apoptosis [22].

Apocynin, an inhibitor of nicotinamide adenine dinucleotide phosphate oxidase (NADPH oxidase [NOX]), has antioxidant properties, protecting the kidneys and heart from the negative effects of oxidative stress [59].

#### 3.2.5. Chronic Inflammation

Inflammation has an important role in both acute and chronic cardiorenal dysfunction. Chronic kidney disease and chronic heart failure are clinical conditions with high levels of circulating inflammatory mediators.

Activation of the endothelium has a direct effect on both inflammation and innate immunity, coagulation, complement activation, and platelet function [60].

The neurohormonal imbalance, along with increased activity of the sympathetic nervous system and the renin–angiotensin–aldosterone system, volume overload, and venous congestion, represent biologic sources of chronic inflammation [61].

Atherosclerosis, the link between chronic kidney disease and cardiovascular mortality and morbidity, is defined by a chronic inflammatory status at the vessel wall level, initiated by endothelial dysfunction. Chronic kidney disease activates vascular inflammatory processes and as consequence high levels of inflammatory biomarkers such as interleukin-6 (IL-6), C-reactive protein (CRP), and tumor necrosis factor (TNF) are associated with an increased risk of myocardial infarction and mortality.

In animal models, it has been demonstrated that oxidative stress, which is present in CKD patients, corresponds with an elevation in inflammatory markers.

Recent clinical trials have shown the advantageous effect of sodium-glucose cotransporter-2 inhibitors in reducing oxidative stress with beneficial cardiovascular and renal effects.

Another condition that could influence the circulating inflammation markers is malnutrition, frequently observed among CKD patients.

Specific therapeutic targets for chronic inflammation are essential for improving patient outcomes.

Inhibition of IL-1β was the interest subject of a randomized, double-blind placebo-controlled trial with over 10,000 patients, where a human monoclonal antibody that targets IL-1β (canakinumab) showed a reduction in recurrent cardiovascular events compared to the placebo [62].

IL-6, one of the most studied cytokines, is associated with the progression of chronic heart failure, representing a predictor of all-cause mortality in this population group. Elevated levels are also present in chronic kidney disease, increasing with the worsening of the CKD stage [61].

Pronounced monocyte activation is present in patients that are hospitalized with acute CRS (type 1 or 3) than hypertensive patients and/or end-stage renal disease [63].

Age, a risk factor for cardiorenal syndrome, is associated with a pro-inflammatory state, representing a risk factor of several age-related pathologies, such as cardiovascular diseases and CKD. Chronic inflammation has an important contribution in both cardiovascular diseases and CKD evolution and progression. Proinflammatory biomarkers such as cytokines and chemokines discharged by the kidneys are implicated in the cardiovascular aging process observed in CKD patients.

High levels of IL-6, TNF-α, and C-reactive protein have been associated with high mortality rates in the elderly. IL-6 has been considered a risk factor for left ventricular hypertrophy in peritoneal dialysis patients [64].

Schunk et al. showed that IL-1α plays an important role in the development of CKD and cardiovascular diseases by regulating tissue accumulation of macrophages and neutrophils, promoting inflammatory injury. Inhibition of IL-1α could represent a future anti-inflammatory treatment target [65].

#### 3.2.6. Other Mechanisms Involved

**Anemia** is a common condition in patients with advanced HF and CKD with a prevalence in CRS from 5% to 55%, reported to be an independent predictor of mortality. Anemia contributes to the pathophysiology of CRS in several ways: can cause tissue ischemia and peripheral vasodilation (leading to activation of SNS), RAAS, the release of antidiuretic hormone (resulting in salt and water retention), vasoconstriction, and chronic renal venous congestion (contributing to progressive interstitial fibrosis and nephron loss). Moreover, the chronic anemic state causes left ventricular hypertrophy [24]. Red blood cells have many antioxidants; therefore, the presence of anemia may cause increased oxidative stress [66].

Gathered evidence suggests that **hyperuricemia** may participate in the appearance and evolution of cardiorenal syndrome. High levels of uric acid are linked to oxidative stress, dysglycemia, insulin resistance, inflammation, cardiac diastolic dysfunction, and renal hyperfiltration—all components of CRS [67]. A low-purine diet along with xanthine oxidoreductase (XOR) inhibitors are part of urate-lowering therapy. Johnson et al. analyzed the use of a recombinant uricase, pegloticase, and obtained a reduction in mean arterial pressure of 5 mmHg in patients with chronic gout and refractory to standard therapy. Other studies have shown the beneficial role of febuxostat in reducing renal disease progression [68].

It is shown that intravenous iron therapy has an important role in the management of anemia in both patients with CKD and HF. Ten percent of patients with cardiorenal syndrome and anemia are guided for erythropoiesis-stimulating agent (ESA) therapy; ESA is not recommended for patients with anemia and HF. Further studies are needed to demonstrate the utility of hepcidin inhibition in the treatment of anemia for patients with cardiorenal syndrome [69].

**Obesity**, through visceral adiposity (which includes intraabdominal visceral fat mass and ectopic fat deposition), contributes to the onset of CRS and is associated with a greater risk for cardiorenal morbidity than subcutaneous adiposity. Obesity can lead to chronic kidney disease through mechanisms such as: hyperfiltration, increased glomerular capillary wall tension, and podocyte dysfunction. This leads to tubulointerstitial fibrosis and a loss of nephrons, cardiac remodeling, and interstitial fibrosis. Heart failure, with a preserved ejection fraction, is associated with obesity-related functional and structural abnormalities [70]. Obesity is considered as heart failure stage A and is associated with an increased aldosterone concentration [71].

Because of the common vascularization of the cardiac and renal system, dysfunction within one system becomes a risk factor of dysfunction in the other [17].

In both patients with heart failure and chronic kidney disease, **gut dysbiosis** has been observed. It is unclear if gut dysbiosis is a potential cause or a downstream effect of cardiorenal syndrome, but one of the incriminated key contributors is uremic toxin accumulation, with cardiac, renal, and vascular effects. To limit internal production of uremic solutes, and to reduce circulating indoxyl sulfate levels, a low-protein diet was beneficial in chronic kidney disease patients along with the use of symbiotic therapy (prebiotic and probiotic) that showed positive modification of the stool microbiome [72].

#### 3.2.7. Biomarkers

In cardiorenal syndrome, biomarkers have become increasingly prevalent in therapy, diagnosis, and prognosis. Studies on biomarkers have just begun and it is very important to note that detailed selection (based on pathophysiological mechanisms for example) (Table 2 and Table 3) and application of these biomarkers remain a challenging process [73].

It has been demonstrated that the severity of CRS is potentially linked to beta-2-microglobulin and tissue inhibitor of metalloproteinases 1 (TIMP 1) levels. Atici et al. found that levels of beta 2 microglobulin correspond to the cardiorenal evolution, proBNP levels (r = 0.66, *p* > 0.0007), with systolic function of the left ventricle (r = 0.56, *p* = 0.0162) and with the glomerular filtration rate (r = 0.83, *p* < 0.0001).

Galectin-3(Gal-3), a promising representant of the beta-galactosidase-binding lectin family, interacts with proteins of the extracellular matrix such as: synexin, integrins, and laminin [7].

Gal-3, after being released by the activated macrophages, induces the activation and deposition of collagen in the extracellular matrix [74,75]. Its principal pathophysiological role, that of promoting fibrosis, can lead, at the cardiac level, to cardiac remodeling and progression of heart failure. Besides this effect, Gal-3 is also involved in renal fibrosis and dysfunction, its greater serum levels being able to precede the decline of GFR [76]. Patients with elevated Gal-3 serum levels showed an accelerated decline of GFR during a three-year follow-up and this was more evident in patients with preserved renal function. In the future, Gal-3, as suggested by some preliminary data, could represent a therapeutic target, not only an indicator for cardiorenal progression [77].

Placental growth factor (PIGF) is another biomarker for CRS status assessment; a member of the vascular endothelial growth factor of cytokines with an important role both in CRS and in CKD alone.

Another intriguing biomarker is urinary cofilin-1, a modulator of epithelial–mesenchymal transition, which is related to the severity of heart failure and is essential for AKI and renal function recovery [7].

A promising approach towards intervention in CRS is the biomarkers that detect changes in collagen turnover in the extracellular matrix of the heart and kidneys. Biomarkers reflecting fibrosis such as Gal-3, NGAL, sST-2, and cardiotrophin-1, may be important in prognostication, early detection, and guiding the treatment of cardiorenal syndrome [78].

In patients with hypertension and kidney injury, biomarkers of cell damage related to systemic oxidative stress such as 8-epi-isoprostanes and plasma thiobarbituric acid-reactive substances are elevated [79,80]. An endothelial injury marker, soluble thrombomodulin, together with angiopoietin-2 can predict acute kidney injury in myocardial infarction patients [81].

Vascular endothelial growth factor (VEGF), platelet-derived growth factor (PDGF), and soluble vascular endothelial growth factor receptors-1 (sFlt-1) are elevated in patients with heart failure. sFlt-1 is a biomarker implicated in endothelial dysfunction in chronic kidney disease and connects microvascular disease with heart failure in CKD.

Another biomarker, a member of the interleukin-1 receptor family, the soluble suppression of tumorigenicity-2 (sST2), is implicated in the development of CKD and is linked with cardiovascular events and survival in CKD patients, being also a tool for prognostic prediction in HF patients with renal dysfunction.

Midregional proadrenomedullin (MR-proADM) is a promising biomarker that predicts the decline of renal function and morbidity in patients with HF [73].

The liver-type fatty acid-binding protein (L-FABP) is an important predictor factor of AKI, with high urinary levels being found in patients with heart failure and AKI.

Arginine vasopressin (AVP) is a peptide with multiple implications in cardiorenal syndrome: causes arteriole vasoconstriction, increases vascular resistance, and promotes water reabsorption in the distal tubule of the kidneys. Several studies are investigating the role of the competitive, selective AVP receptor antagonist, tolvaptan, in cardiorenal syndrome. Copeptin, the C-terminal portion of the AVP, was shown to be associated with cardiovascular disease in patients with CKD [7].

## 4. Future Perspective

In cardiorenal crosstalk, the essential aim is to consider that patients with cardiorenal syndrome are not homogeneous. There are five types of cardiorenal syndromes, but billions of patients with different pathophysiological characteristics need individualized approaches.

Future studies and trials are necessary to explore the one common and major pathological pathway responsible for renal and cardiac injury before the onset of symptoms. Therefore, the field of biomarkers continues to be a challenge, especially in type 5, where a systemic condition simultaneously produces cardiac and renal dysfunction.

## 5. Conclusions

The complex pathophysiology of CRS remains a challenging field. The complex interaction between the heart and kidneys continues to open new pathways for discovering and exploring possible mechanisms. In order to improve diagnostic processes, treatment strategies, and prognostic for patients with cardiorenal syndrome, focusing on the pathophysiological process and biomarkers represent a warranted tool.

Despite being a diagnostic key, biomarkers provide important prognostic information; their serum level has a direct impact on mortality. Understanding cardiorenal syndrome from endothelial dysfunction, fibrosis, and inflammation to epigenetics is essential and future studies are needed to encompass this vast pathology.

## Figures and Tables

**Table 1:** Cardiorenal syndromes classification.

Cardiorenal Types	Known as	Characteristics and Mechanisms	Etiology/Clinical Conditions
Type 1	Acute cardiorenal syndrome	Acute worsening of cardiac function cause acute kidney injury (AKI)	Acute ischemic heart disease, cardiogenic shock, acute decompensated heart failure
Type 2	Chronic cardiorenal syndrome	Chronic cardiac dysfunction leading to progressive renal impairment	Chronic heart failure
Type 3	Acute renocardiac syndrome	Acute kidney injury leads to acute cardiac dysfunction	AKI, uremia, kiperkaliemia, volume overload leading to pulmonary edema
Type 4	Chronic renocardiac syndrome	Chronic kidney disease contributes to decreased cardiac function	CKD-associated cardiomyopathy
Type 5	Secondary cardiorenal syndrome	Acute/chronic systemic condition leading to cardiac and renal impairment	Diabetes mellitus, sepsis, amyloidosis, cirrhosis

AKI: acute kidney injury; CKD: chronic kidney disease.

**Table 2:** Pathophysiological mechanisms along with biomarkers in cardiorenal crosstalk.

	Pathophysiological Mechanism	Biomarkers
**Hemodynamic pathways**	Cardiac output	BNP, copeptin, cardiac troponin-I
	Arterial pressure	CRP, TNF alpha, ox-LDL,
	Extracellular fluid volume	NT-proBNP, cardiac troponin-I, copeptin
	Elevated renal venous pressure	Creatinine
**Non hemodynamic pathways**	Fibrosis	Gal-3, NGAL, sST-2, cardiotrophin-1
	Oxidative stress	MMP, Ox-HDL, MR-proADM, 8-epi-isoprostanes
	Obesity	Aldosteron
	Endothelial dysfunction	sFLT-1, VEGF, PDGF soluble thrombomodulin, angiopoietin-2, anti-endothelial cell antibodies
	Chronic Inflammation	C-reactive protein, procalcitonin, NGAL, IL-6, IL-18, TNF-alpha
	Epigenetics	microRNAs, miR-21

BNP: B-type natriuretic peptide; NT-proBNP: N-terminal pro-brain natriuretic peptide; CRP: C-reactive protein; Gal-3: Galectin-3; sST-2: soluble suppression of tumourigenicity 2; MMP: matrix metalloproteinase; Ox-HDL: oxidized high-density lipoprotein; sFlt-1: soluble vascular endothelial growth factor receptors-1; VEGF: vascular endothelial growth factor; PDGF: platelet-derived growth factor; IL-18: interleukin-18; IL-6: interleukin-6; MR-proADM: midregional proadrenomedullin; NGAL: neutrophil gelatinase-associated lipocalin; miR-21: microRNA 21; TNF-alpha: tumor necrosis factor-alpha.

**Table 3:** Biomarkers associated with cardiorenal syndrome types.

Cardiorenal Type	Biomarkers
**1**	BNP, NT-proBNP, creatinine, cystatin C, KIM-1, NGAL, MR-proADM, IL-6, IL-18,
**2**	BNP, NT-proBNP, creatinine, cystatin C, microalbuminuria, aldosterone, miRNA-21,NGAL, KIM-1
**3**	BNP, NT-proBNP, creatinine, cystanin C, NGAL, KIM-1, netrin-1, IL-6, IL-18,
**4**	BNP, NT-proBNP, creatinine, cystatin C, troponins, CRP, homocysteine, uric acid, microalbumineria, aldosterone
**5**	BNP, creatinine, procalcitonin, CRP, IL-6, TGF-beta, Gal-3, sST2

BNP: B-type natriuretic peptide; NT-proBNP: N-terminal pro-brain natriuretic peptide; NGAL: neutrophil gelatinase-associated lipocalin; KIM-1: kidney injury molecule 1; miRNA-21: microRNA 21; IL-18: interleukin-18; IL-6: interleukin-6; Gal-3: Galectin-3; CRP: C-reactive protein; sST-2: soluble suppression of tumourigenicity 2; TGF-beta: transforming growth factor beta; MR-proADM: midregional proadrenomedullin.
